# Ionic Liquids as Antifungal Agents for Wood Preservation

**DOI:** 10.3390/molecules25184289

**Published:** 2020-09-18

**Authors:** Catalin Croitoru, Ionut Claudiu Roata

**Affiliations:** Materials Engineering and Welding Department, Transilvania University of Brasov, Eroilor 29 Blvd., 500039 Brasov, Romania

**Keywords:** ionic liquids, wood, antifungal character, fungicides, preservatives, alkylimidazolium, alkylammonium

## Abstract

Ionic liquids represent a class of highly versatile organic compounds used extensively in the last decade for lignocellulose biomass fractionation and dissolution, as well as property modifiers for wood materials. This review is dedicated to the use of ionic liquids as antifungal agents for wood preservation. Wood preservation against fungal attack represents a relatively new domain of application for ionic liquids, emerging in the late 1990s. Comparing to other application domains of ionic liquids, this particular one has been relatively little researched. Ionic liquids may be promising as wood preservatives due to their ability to swell wood, which translates into better penetration ability and fixation into the bulk of the wood material than other conventional antifungal agents, avoiding leaching over time. The antifungal character of ionic liquids depends on the nature of their alkyl-substituted cation, on the size and position of their substituents, and of their anion. It pertains to a large variety of wood-colonizing fungi, both *Basidiomycetes* and *Fungi* *imperfecti*.

## 1. Introduction

Wood represents one of the oldest natural materials, still widely used to date (circa 10,000 different end-products) due to its optimized strength per weight ratio, high workability, and aesthetic appeal [1,2]. Due to its high carbohydrate content (up to 70 wt.% cellulose and hemicellulose), it is particularly susceptible to fungal attack, which is one of the main causes of wood loss (both of wood biomass as well as wood end-products) [3,4,5]. The impact of fungal degradation usually ranges from changes in appearance (modification of natural wood color) to drastic changes in mechanical properties and dimensional stability, in most cases rendering wood completely useless [6,7]. Provided that the temperature (generally 10 to 30 °C) and wood equilibrium moisture (higher than 20%) conditions are right, without preventive measures being taken, most wood materials generally have a lifecycle of fewer than three years (included in the class IV and V of durability) [8]. Only a limited range of exotic wood species are inherently resistant to fungal attack, with an end-product lifecycle of more than eight years (class I of durability). However, the widescale use of these types of wood is not economically sustainable [8,9].

One of the most efficient methods for fungal attack prevention (aside from chemical functionalization, impregnation with synthetic resins or coating methods, e.g., painting) is represented by the passive or pressure impregnation with different types of preservatives [10,11]. Historically, oil-based preservatives (including those containing polycyclic aromatic hydrocarbons) were among the first used [12], followed by waterborne preservatives based on silicates, borates, fluorides, heavy metal ions salts (especially Zn^2+^ and Cu^2+^), metallic or metal oxide particulate dispersions, metal-heterocyclic compounds complexes, or salts of arsenic/arsenous acid [13,14,15]. These formulations generally represent efficient antifungal formulations in terms of considerably extending the lifecycle of wood end-products on a mid or long-term level, but possess significant shortcomings such as relatively high toxicity (aside from silicates and some borates), and a moderate to high leaching potential [16,17,18]. Also, these “traditional” preservatives raise additional issues when recycling/disposing of the preservative-impregnated wood [19]. The use of one of the most benign wood preservatives, chromated copper arsenate (CCA) was heavily restricted by the European Commission in 2003 and by US Environmental Protection Agency in 2004 due to its toxicity and high susceptibility of soil and groundwater buildup [20].

**Figure 1:**
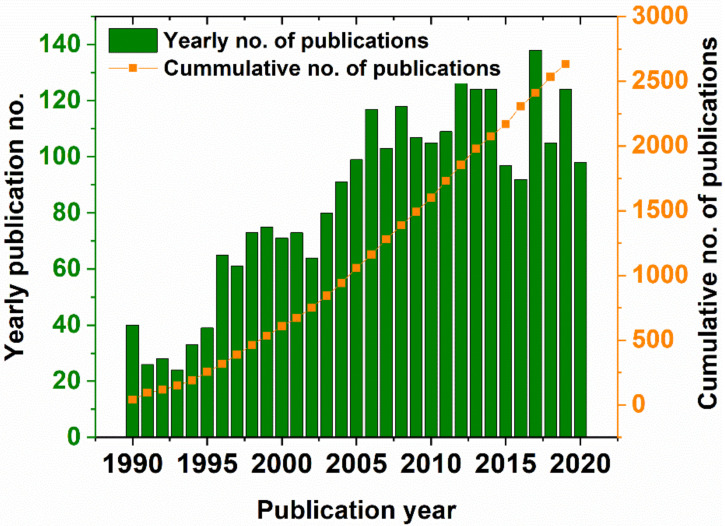
Yearly and cumulative number of publications in the domain of wood preservatives in the 1990–2020 period (Database: Scopus, search terms: “wood” AND “preservative~”, search date: 18 August 2020).

Incidentally, if one looks at the cumulative number of papers on the subject of wood preservation (Figure 1), the year 2004 represents a turning point in the number of published papers, with a steady increase (of ~100 papers per year) in the interest related to this research subject. Conversely, the emphasis is placed on methods of wood preservation by compounds that have a high ratio of antifungal efficiency per environmental and economic impact.

Many alternative natural or bio-inspired fungi-active products are continuously devised, based on natural plant extracts, such as, for example, essential oils (terpenes) [21], wood extractives (e.g., tannins) [22,23], biopolymers [24,25], oils [26], either standalone or in combination with “classical” antifungal compounds. These formulations present debatable economic efficiency and scalability potential. As such, the wood preservation topic is still open and dynamically expending, continuously reporting new alternative treatments.

One class of emerging wood preservative candidates could be ionic liquids (ILs). These ionic organic compounds, which have been around since the late 1970s, have been intensively researched in the last decade concerning their solvation ability for lignocellulose, biomass derivatization potential, and multipurpose-additives for wood-based materials (antistatic agents, thermal and radiation stabilizers, plasticizers, and so forth) [27,28,29,30].

To date, there are less than thirty research papers related to the use of IL as antifungal agents for wood (not counting those related to cellulose and paper), which is very few, considering the high synthesis versatility of this class of compounds (there are mainly nine types of “conventional” organic cations, with a practically unlimited choice of anions). The advantages of using ILs as preservatives reside in their excellent absorption and fixation into the wood’s structure without altering its texture or color [30,31], and their low vapor pressures, which account for low overall atmospheric emissions [32]. Also, due to their ability to swell wood, they could be used as carriers for different antifungal agents or as means for several moieties with antifungal character grafting on their lateral alkyl chains [33,34]. However, the use of ILs as fungicides is not without problems: in some cases, their prices can be prohibitive, and their long-term toxicity profiles are not yet fully assessed, but for some particular cases, existent studies seem to indicate that the benefits could long outweigh these shortcomings [35]. For example, some long-chain alkoxyalkylpyridinium and alkylammonium ILs could be synthesized using cost-efficient raw materials and some efficient antifungal agents, such as triazolium ILs present a very low toxicity in relation to animals (acute oral LD_50_ for female rats exceeds 4000 mg/kg), which makes them less toxic than common table salt [36,37].

This review paper is dedicated to assess the use of ionic liquids as wood preservatives and to establish several correlations between the chemistry of the ILs, their effect on the lignocellulose material and their antifungal activity. This paper would be the first standalone review study on this research topic. This review does not advocate the replacing of the well-established wood preservation agents with ILs, nor claim that ILs are better. It presents the relatively little data available from the reference literature in a critical manner, highlighting both the advantages and disadvantages of this versatile class of organic compounds in this particular niche domain.

## 2. Wood Structure and Composition

Wood is a complex material, a natural composite comprised of carbohydrate polymers (cellulose, hemicellulose, and pectins), lignin (a heterogeneous aromatic polymer), glycoproteins, and minor amounts of small molecular weight chemicals (extractives), assembly often being called lignocellulose [38].

The outermost layer of the wood cell wall is the middle lamella, which is mainly comprised of pectins and acts as a binder between neighboring cells. Cellulose is the main component of wood, and its long molecules associate with each other through hydrogen and van der Waals bonds to form supramolecular structures called cellulose microfibrils. Microfibrils are associated through intramolecular bonding and intermolecular bonding with other carbohydrates, mainly hemicellulose and pectins, which result in fibrils and cellulose fibers, constituting the primary cell wall of wood cells. The primary cell wall is composed of 25–30% of cellulose, 30% of hemicellulose, 35% of pectins, and 1–5% of glycoproteins (as dry weight percentage) [39,40].

The wood cells used as support develop an additional wall layer called the secondary cell wall. It is deposited between the plasma membrane and the primary cell wall. One particularity of the secondary cell wall is that it forms a large part of the cell wall mass in wood and contains lignin, which binds together the microfibrils and ensures high rigidity of the microfibrillar assembly. In some wood cells, three layers can be distinguished in the secondary cell wall: S1, S2, and S3, each with a different orientation of its fibers of cellulose. The secondary cell wall is usually interrupted by pores or pits, which allow neighboring cells to communicate. The secondary cell wall is composed mainly of cellulose (40–60% of the dry weight), hemicellulose (10–40% of the dry weight) and lignin (10 to the 35% of the dry weight) [38,40].

## 3. Short Overview on Fungal Degradation Mechanisms of Wood

Wood decay by fungi is typically classified into three types, based on the overall physical and morpho-structural characteristics of the attacked wood (Table 1): brown rot, white rot, and soft rot. Every kind of rot is specific in terms of the degradation mechanisms employed for the main components of the wood cell wall (cellulose, hemicellulose, respectively lignin) [41,42]. Due to their intricate complexity, these mechanisms are not entirely understood, but their basic outlines have been elucidated and may help to develop further efficient strategies for antifungal protection [43].

Both brown and white rot fungi use low molecular weight mediators in their earliest stages of wood colonization [45]. Examples of such mediators are Fe^2+^ and H_2_O_2_ (similar to a Fenton process, which produces highly-reactive hydroxyl radicals) and/or glycopeptide(s). These mediators promote oxidation and depolymerization of hemicellulose, which leads to “de-bonding” of lignin from the cellulose microfibrils [46,47]. The other wood constituents are also degraded to some extent at this stage. Oxidation starts from the amorphous regions of the microfibrils and from the chain end units in the case of cellulose, respectively from the side chains in the case of lignin. The initial release of mediators provides the pathway for the in-depth penetration of the fungal hyphae and the diffusion of specialized enzymes into the structure of the wood at more advanced stages of colonization. These enzymes are used to destructure and depolymerize cellulose, hemicellulose (brown rot, simultaneous white rot, soft rot) or lignin (simultaneous white rot, selective white rot) [46,47,48,49].

**Figure 2:**
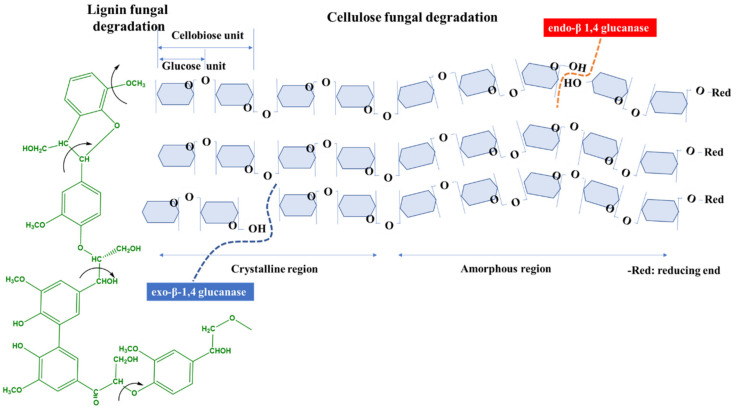
Schematic depiction of lignin and cellulose chain scission induce by fungal enzymes.

Brown rot fungi, soft rot fungi and some simultaneous white rot fungi are known to produce endo- or exoglucanase (“cellulases”), which can cleave the β-l,4-glucosidic linkages from cellulose producing a wide range of oligosaccharides, ranging from dextrins to cellobiose. Finally, β-glucosidase hydrolyzes cellobiose or other short oligosaccharides to glucose, which can be readily used by the fungus. Exo-β-1,4 glucanase removes single cellobiose or glucose units from the non-reducing end of the cellulose chain. In contrast, endo-β 1,4 glucanase cleaves cellulose at random sites along the chain, but preferentially from the amorphous regions (Figure 2) [50,51,52].

White rot fungi and some species of soft rot fungi can secrete both endo- or exoglucanases, while brown rot fungi secrete only endoglucanase, the latter being less efficient in cellulose metabolization [44,46]. Similarly, “hemicellulases” are employed by all types of wood-degrading fungi to cleave hemicellulose [41,53].

White rot fungi can produce an extensive enzymatic system that can completely degrade wood, not only the polysaccharide components (simultaneous white rot fungi) but also lignin (selective white rot fungi). The enzymes involved in this ligninolytic activity include lignin peroxidase (which can oxidize both phenolic and non-phenolic structural units of lignin, Figure 2), manganese peroxidase (which is based on the Mn^2+^→Mn^3+^ redox system), and laccase [46,54,55].

Laccase can also be used to degrade lignin completely, but only in the presence of specific mediator fungal metabolites. This type of enzyme can also be produced by some species of soft rot fungi, which may have an attack pattern similar to selective white rot fungi [55,56]. At any stage, fungal colonization can be followed by the installment of bacteria, molds, or other pathogens, which could further extend the damage on wood [57].

## 4. Antifungal Wood Preservation

**Figure 3:**
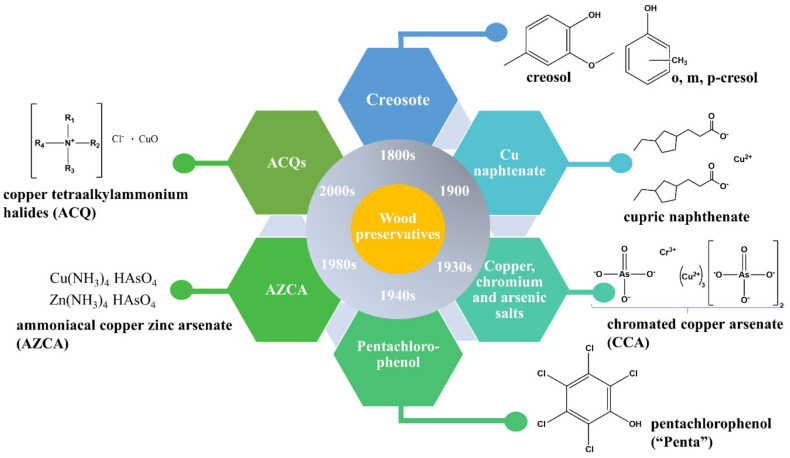
Overview scheme of the main types of wood preservatives used to date.

Treatment of wood structures with preservatives leads to an increase in their lifetime, reducing replacement costs, and allowing for more efficient forest biomass use. There are numerous classes of antifungal agents, ranging from fossil fuel and biomass distillates (creosote), which are among the longest in service, to tetraalkylammonium halides (the so-called “quats”) (Figure 3). “New” generation preservatives include alkaline copper quaternary compounds (ACQs), waterborne cupric naphthenate (CuNap-W), azoles, and copper azoles (CAs), oligomeric alkylphenol polysulfides (PXTS), bis copper-8-quinolinolate (Ox), and so forth [55]. The distinction between “old’ and “new”-generation preservatives is strictly made based on the decades they have emerged in industry-scaled applications and does not imply that they are presently not used anymore. On the contrary, the “long-runners” creosote and pentachlorophenol still make up about 20% of the total market share for wood preservatives [58,59].

Four essential prerequisites must be met for a formulation to be deemed as efficient in terms of wood preservation: (i): they must present effective doses (ED) respectively lethal doses (LD) as low as possible for the intended type of wood and fungi strains; (ii): they must present good penetrability into the wood and low leaching potential; (iii): they must possess low environmental and health risks and (iv): they must be economically efficient [60].

While creosote, pentachlorophenol and AZCAs are strictly regulated and used only in limited instances, ACQs (with halide and carbonate anions), CuNap-W and CAs are currently approved for full exposure to above ground, ground contact, and freshwater applications and represent ~50% marketshare of the total wood preservative industry. These formulations can be used as standalone or in combination with borates or copper salts. However, ACQs present somewhat limited antifungal efficiency, usually being combined with other fungicidal compounds, and CAs are expensive [60,61].

There are many formulations studied for their efficiency as wood preservation. One of the promising research directions would be the use of nanometric metal particles. Screening studies have shown that a 1 wt.% dispersion of 30 nm Cu, Zn, and B nanoparticles significantly inhibits the growth of brown rot fungi, while completely eradicating other non-fungal wood pests (termites, molds). Metal oxide nanoparticles (e.g., ZnO) are less effective as fungicides [62].

There are currently three strategies of wood preservation, which can independently or simultaneously be involved in providing antifungal resistance to wood: biochemical, chemical, and physical [63,64], summarized in Table 2. 

By far, the most efficient and complicated route of wood preservation is the biochemical one, providing the preservatives have a low leaching potential over time. Many commercial preservatives (including creosote, quaternary ammonium salts, azoles or Cu^2+^ salts, for example) and plant extracts can disrupt both fungal growth and cellulase enzymes secretion, presenting an evident biocidal (antifungal) character [80,81,82]. The biochemical strategy implying non-recognition of specific substrates by the cellulolytic and ligninolytic enzymes has been proven only to slow down fungal degradation, not completely inhibiting it [64]. The same could be said concerning the physical means of protecting wood through voids filling (e.g., through impregnation with synthetic resins). The preservation agents acting through the biochemical pathway generally do not affect the mechanical properties of wood, except for long-chain alkylammonium compounds, which may act as plasticizers, decreasing wood hardness. In some particular cases, e.g., for creosote or Cu^2+^ salts, the dimensional stability of wood to moisture can be improved [70].

The chemical route has been lately proven as imparting lower durability to wood compared to the biochemical one. For example, the chemical mode of action is efficient if the moisture of wood is kept relatively low. Furthermore, many antioxidant compounds of natural origin are relatively volatile, needing auxiliary methods of permanent fixation (i.e., physical, by pores and voids filling) [83].

Physical methods implying moisture exclusion (thermal treatment at 120–150 °C, chemical crosslinking) can be almost as efficient as their biochemical counterparts and represent one of the most economically efficient means of wood preservation. The advantage of these methods relies on imparting multiple beneficial properties to wood [84,85]:

- antifungal resistance (if the thermally-treated wood is crosslinked, i.e., moisture stabilized)
- insect and molds resistance
- improved dimensional stability and high hardness

Since thermal treatment also partly degrades some wood components (extractives, hemicellulose, cellulose), there may be unwanted modifications in the color of wood and 5 to 20% reduction in the modulus of rupture [86].

## 5. Ionic Liquids Applications Related to Wood

**Figure 4:**
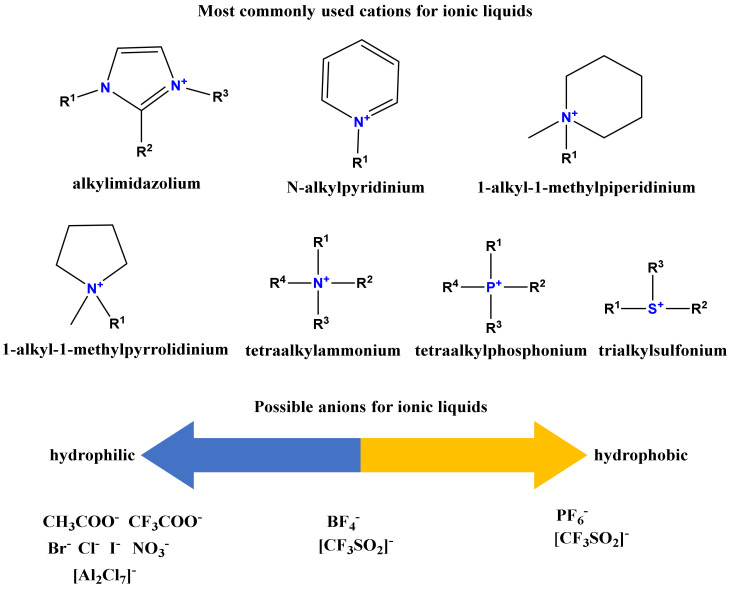
Possible cations and anions for ionic liquids.

Ionic liquids (also known as designer solvents, ionic fluids, or molten salts) are organic salts with melting points below that of water at ambient pressure. To be liquid at room temperature, ILs must preferably present asymmetric cations (i.e., alkyl side chains with different sizes) [87]. The main types of cations encountered in the structure of most frequently used ILs are presented in Figure 4.

The insolubility of wood’s main components (cellulose, hemicellulose, and lignin) in common solvents has long been hampering the development of new methods for efficient utilization of wood products [88]. Due to their high synthesis derivatization potential arising from a large variety of cation and anion combinations, ionic liquids could be used in various ways related to wood:

- as wood pulping and fractionation media, through the selective dissolution of wood components and later precipitation with a non-solvent, such as water. Short-chain (<C4) ionic liquids (especially those with 1-*n*-alkyl-3-methylimidazolium cation and chloride/acetate anion) present good cellulose dissolution ability. In contrast, those with long-chain side alkyl chains or aromatic substituents (especially when combined with bulky anions such as acetate or acesulfamate) are more selective towards lignin, possessing low cellulose dissolution ability. 1-ethyl-3-methylimidazolium formate is highly selective towards the dissolution of hemicellulose [89,90];
- as pretreatment media for wood biomass to improve the accessibility of the cellulase enzymes in the bulk of the wood [91];
- as media for the cellulose component chemical modification, e.g., acetylation of wood in and 1-ethyl-3-methylimidazolium chloride/acetate mixture [92];
- as media for obtaining useful chemicals through depolymerization of wood lignin (5-hydroxymethylfurfural can be obtained after a long-run 24 h thermal treatment of wood 1-ethyl-3-methylimidazolium chloride) or wood cellulose (maltose, nigerose, kojibiose, laminaribiose, isomaltose, and gentiobiose can be obtained from cellulose through treatment with 1-ethyl-3-methylimidazolium chloride) [93,94];
- as carriers for different impregnants or antifungal agents into the structure of wood. In this application, ILs are useful through improving the penetration ability of the transported species through promoting wood swelling [25,33];
- as plasticizers for wood: ionic liquids with alkylammonium, alkylpyridinium or 1-*n*-alkyl-3-methylimidazolium (>C10) cations, do not dissolve the wood components, but can disrupt the inter- and intramolecular bonding in cellulose and/or lignin and increase the number of amorphous domains in opposition with the crystalline ones [30,95];
- as additives, providing various functionality: fire retarders (especially alkylphosphonium ILs, or ILs with hexafluorophosphate or tetrafluoroborate anions) [96], thermal and UV stabilizers [29,97], antistatic agents [98] and so forth.

Despite their promising results in correlation to wood processing and additivating, ionic liquids are not “problem-free”. Due to trace amounts of impurities (<0.1 wt.%), unwanted cellulose hydrolysis could occur, as well as cellulose and lignin thermal and chemical degradation at temperatures higher than 110 °C. Moreover, due to their very high viscosity (typically 100–2000 *cP* at room temperature), wood additivating with these compounds usually implies ILs dissolution in a carrier (water or ethanol) or high temperatures, the latter which can promote unwanted modifications in wood [99].

## 6. Antifungal Character of Ionic Liquids

Ionic liquids can act as antifungal agents through all modes of action (biochemical, chemical and physical), although a clear description of their detailed antifungal mechanism is lacking in the reference literature. Based on the ILs antibacterial and anti-mold mode of action, both more researched, it is likely that heterocyclic ILs (especially alkylimidazolium, alkylpyridinium) can disrupt the synthesis of ergosterol, an important cell wall component, similar to azole compounds. Also, ionic liquids (even long-chain hydrophobic ones) can actively interact with proteins or polysaccharides from the fungal hyphae cell walls [100,101]. Low molecular mass ionic liquids could act as non-targeted chaotropes, destructuring and disrupting the tertiary buildup of proteins (including enzymes) or polysaccharides [102].

Based on studies on UV or electron-beam irradiated wood treated with ionic liquids, it has been demonstrated that ILs are involved in free radicals trapping, thus protecting the wood against deterioration. The same mechanism could be involved for the free-radical fungi-induced deterioration of cellulose and lignin [29,103].

Hydrophobic ILs (which are either oily viscous liquids or wax-like solids) can lower the surface energy of wood, moisture content, and water absorption, inhibiting the spreading of the degrading agents. These facts seem to imply that ionic liquids with longer side chains (and higher molecular mass) are more efficient either in inhibiting fungal growth (fungistatic character), as well as in fungal colonies complete neutralization (fungicide character) [104]. The experimental data available to date seems to confirm this trend, i.e., the antifungal activity is proportional to the IL hydrophobicity [105,106]. However, after 6–12 C atoms/side chain (not counting the C atoms involved in aromatic or aliphatic cycles), an inhibiting effect in the efficiency of the ILs have been observed. The optimum molecular mass of the cation (i.e., size of the lateral alkyl chains) is strongly dependent on the type of cation base (e.g., ammonium, imidazolium, and so forth) and on other short-sized substituents. This limiting effect could probably be due to lower mobility of these ILs and their limited interaction with the fungi or enzymes (van der Waals bonding) and their effective charge screening by the large alkyl substituents [36,107].

Also, it has been observed that for protic ionic liquids, the increase of the alkyl chain length in the cation decreases their surface tension up to a critical value (C8–C12), above which any further increase leads to an increase in the surface tension, lowering their antifungal efficiency. The optimal biological effect at a specific cation chain length is regularly due to a combination of hydrophobicity, absorption capacity, and critical micelle concentration (CMC). Higher molecular weight ionic liquids tend to be fixed better in the structure of wood, with minimal leaching potential [108].

More hydrophobic or bulkier anions associated with the same cation tend to be more efficient in impairing fungal growth or eradicating the inoculums. ILs with lower molecular mass cations could even penetrate the fungal cell wall (similar to the traditional azole or polyene compounds) driven by diffusional and osmotic gradients. Still, these tend to necessitate a higher critical concentration to be effective. The higher amount needed for low molecular weight ILs to be effective could be due to their higher absorption into the wood cell walls/ microfibrils, only a low amount remaining at the surface to interact with the fungal hyphae or with the secreted enzymes [108,109].

Usually, pairing low molecular mass cations with bulkier and more hydrophobic anions increases their antifungal efficiency, but the cation’s molecular mass represents the dominant influence. The ILs with low molecular weight cations tend to leach more readily from wood, but come in very useful as carriers for other fungicidal compounds, because of their wood swelling ability [110].

**Figure 5:**
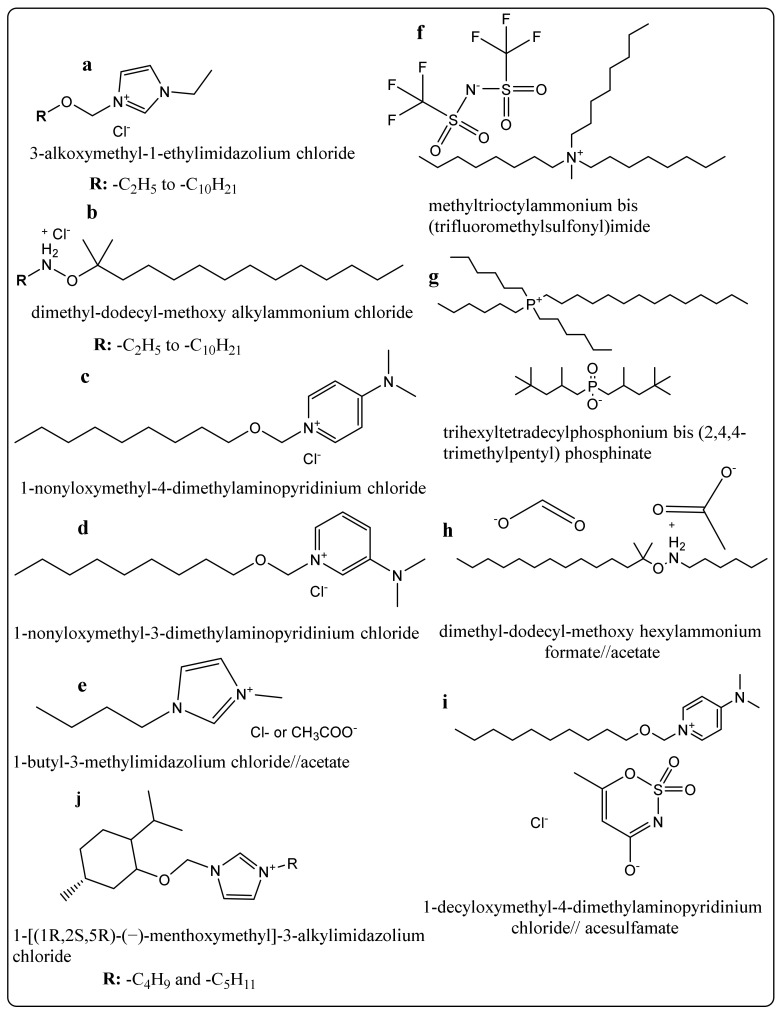
Chemical structures of the ionic liquids with antifungal character.

One of the first studies dedicated to the fungicidal action of ILs studied the influence of 3-alkoxymethyl-1-ethylimidazolium chlorides (Figure 5a) of different molecular masses on three types of wood-degrading fungi: *Coniophora puteana* (white rot fungus), *Trametes versicolor* (brown rot fungus) and *Chaetomium globosum* (soft rot fungus). The results have indicated that the increase in size for the lateral alkyl side chains from -C_2_H_5_ to -C_10_H_21_ determines an exponential decrease with up to 95% in the ED_100_ (preservative concentrations retarding the fungal growth rate by 100% in comparison with the reference) and LD (concentration causing the death of inoculum) values for all three types of fungi. Imidazolium chlorides with larger alkyl substituents (e.g., -C_12_H_25_) exhibited lower antifungal efficiencies [111]. For ammonium ionic liquids (of the type dimethyl-dodecyl-methoxy alkylammonium, Figure 5b), the different alkyl substituents’ optimum size was C6-C7. For these ammonium ILs, a strong polynomial correlation has been found between the LD and EC_100_ values against *Chaetomium globosum* and their CMC [112].

The presence of bulkier substituents in the side chains of the cation (such as cycloalkyl or phenyl) enhances the antifungal character of the ILs. For example, for imidazolium chloride ILs, the highest antifungal activity was registered for cyclooctyl and cyclododecyl substituents [112]. Similarly, halogen or oxygen heteroatoms in the side chain or the presence of sp^2^ double-bonds (such as in the allyl radical) tend to enhance the biocidal effect, due to a better interaction with the fungal cell membrane [32,108].

The position of the substituents in heterocyclic IL cations (e.g., pyridinium, for which there is quantitative data available) can also be correlated to the ILs antifungal character. For example, the LD values are 20% higher for 1-nonyloxymethyl-4-dimethylaminopyridinium chloride (Figure 5c) versus 1-nonyloxymethyl-3-dimethylaminopyridinium chloride (Figure 5d) against *Sclerophoma pityophyla* (a wood staining filamentous fungus), while for *Trametes versicolor*, respectively *Coniophora puteana* there is a non-significant difference in these ILs antifungal activity [113].

The choice of anions greatly influences the antifungal activity of ionic liquids. When comparing the antifungal character of 1-butyl-3-methylimidazolium chloride (BC) and 1-butyl-3-methylimidazolium acetate (BA) (Figure 5e) against *Chaetomium globosum* (soft rot fungus), it can be seen that BA presented a fungal inhibition potential up to 90% higher than BC, but still 1000 lower than the antifungal drug clotrimazole [114]. A study on the effect of various imidazolium ionic liquids on human pathogenic filamentous fungi has found a direct proportionality between the hydrophobicity of the anion and its antifungal character. For the same type of cation, the antifungal character increases in the order ${\mathrm{Cl}}^{-}$ << ${\mathrm{BF}}_{4}^{-}$ << ${\mathrm{PF}}_{6}^{-}$, alongside with hydrophobicity (lipophilicity) [115].

Anions with a basic Lewis character such as carboxylates, bis (trifluoromethylsulfonyl)imide or bis (2,4,4-trimethylpentyl) phosphinate could possess a higher susceptibility for interaction with the fungal cell wall, with fungal proteins, or with the ions involved in the non-specific or enzymatic wood degradation step (Fe^2+^, Mn^2+^) [116,117,118].

Scots pine wood (*Pinus sylvestris*) treated with protic alkylimidazolium, and alkylmethoxyimidazolium ionic liquids with lactate and salicylate ions present good antifungal activity against *Sclerophoma pityophila* (a parasitic fungus than colonizes the needles of coniferous species). Still, there is no available data to compare their efficiency with the corresponding chloride analogs. The ionic liquids methyltrioctylammonium bis (trifluoromethylsulfonyl)imide (Figure 5f) and trihexyltetradecylphosphonium bis (2,4,4-trimethylpentyl) phosphinate (Figure 5g) can completely inhibit the development of *Postia Placenta* (brown rot) inoculum after three days of exposure. It can be used as a preservative either for wood alone or for wood composites [117].

For ammonium ionic liquids, the anion seems to have a more pronounced influence on the antifungal character. There is a 100% increase in the ED_100_ value against *Chaetomium globosum* when comparing dimethyl-dodecyl-methoxy hexylammonium formate with dimethyl-dodecyl-methoxy hexylammonium acetate (Figure 5h). Didecyldimethylammonium ionic liquids paired with different types of anions: nitrate, nitrite, 4-chloro-2-methyl-phenoxy acetate, and 3-aminotriazolate anions show an increasing fungitoxic efficiency against *C. puteana* in the order nitrate << 3-aminotriazolate < 4-chloro-2-methylphenoxyacetate ≈ nitrite [36].

For other types of cations, e.g., pyridinium, the anion has a lower influence on the antifungal character. For example, the doses and toxic values for 1-decyloxymethyl-4-dimethylaminopyridinium chloride vs. acesulfamate (Figure 5i) were practically the same against all the tested fungi strains (*Sclerophoma pityophyla*, *Coniophora puteana*, and *Trametes versicolor*). They were comparable with benzalkonium chloride and didecyldimethylammonium chloride, two commonly used “traditional” wood preservatives [113].

Introducing antifungal moieties into the side alkyl chains of ionic liquids represents a new strategy to tune the efficiency of these compounds as wood preservatives. An example is introducing an optically-active (1*R*,2*S*,5*R*)-(−)-menthoxymethyl substituent into the structure of butylimidazolium and pentylimidazolium chlorides(Figure 5j), allowing for the obtaining of chiral ILs, with fungicidal properties ranging from 1.67 to 2.85 kg m^−3^ against *Coniophora puteana* and *Chaetomium globosum*. However, the antifungal properties of these ILs were lower than benzalkonium chloride and didecyldimethylammonium chloride, and even than that of pure menthol [34]. It seems that grafting the optically active moiety to the ionic liquid induces some steric hindrance, so the grafted chiral unit is less efficient as a biocide in the IL than the standalone compound. There is no available experimental data to conclude if there is an optimal type of cation base to which a chiral moiety could be added so that it could confer the maximum possible fungicidal effect. Studies on different bacteria and mold strains indicate a biocidal effect decreasing in the order ammonium > alkylimidazolium > alkoxymethylimidazolium > pyridinium (for chloride ILs and grafted menthol chiral moiety) [119].

Evidently, not all optically-active moieties appended to an IL have antifungal character. For example, didecyldimethylammonium and benzalkonium D, L- and L-lactates have been shown to possess low wood antifungal properties [120].

Ionic liquids can also be used as carriers (solvents) for a wide variety of antifungal compounds, ranging from small-molecular ones (e.g., azoles, alone, or in combination with copper salts) to macromolecules (e.g., chitin or chitosan) [121,122]. Organic ionic liquids incorporating cations derived from the antifungal drug miconazole (such as tebuconazole or propiconazole with a halide or citrate anions) could be used alone or in combination with alkylammonium ionic liquids to protect wood against a large variety of *Basidiomycetes* and filamentous fungi [36,123].

Due to their high solvation ability, ionic liquids could also serve as extraction and/or derivatization media for natural biocidal compounds, which could possess activity against pathogenic wood fungi as well (e.g., terpene-containing essential oils, heterocyclic compounds, tannins, polyphenols and so forth [25,124].

## 7. Future Perspectives

In our opinion, future development in the domain of IL preservatives should exploit the excellent ability of ILs to act as complexing agents for heavy metal cations or as carriers for various inorganic and organic compounds and structures. Many studies have reported that Cu^2+^ addition (in azole or borate-based formulations, for example) greatly enhances their antifungal character. Ionic liquids could have superior penetration abilities and could aid in a prolonged retention into wood, increasing its service life. Anions containing transitional metals (such as tetrachlorocuprate or tetrachloroferrate) are already available in conjunction with alkylpyridinium or alkylimidazolium cations [125]. Ionic liquids could also serve as a good synthesis and dispersion medium for nanoparticles and could be used as carrier for the impregnation of wood, potentiating the antifungal effect of the metal nanoparticles [126].

Another cost-efficient and unexploited direction that could be followed is the use of ILs with “sweet” cations (i.e., derived from carbohydrates). These could efficiently act through the biochemical mode of action altering the cellulase-glucose feedback loop or could be absorbed by the fungal hyphae, leading further to destructuring of the fungal cells.

Ionic liquids are expected to account for a total market share of 22 billion US dollars in 2022, as solvents and derivatization media. Their implementation in wood-related applications is mainly hindered by their high cost. If in 2010, 1-ethylimidazolium chloride of 95% purity cost about €720 per kg, today the price has dropped to €435 per kg, and it is expected that it will continue to lower, as more large-scale companies will be involved in their mass production. Still, alkylimidazolium chlorides (among the most commonly used ILs) are 1000 times more expensive than traditional solvents employed for wood finishing (ethanol, acetone, dimethylsulfoxide) or preservatives used for wood impregnation [99,127].

However, the results have indicated that ILs tend to have a lower LD value than traditional wood preservatives such as tebuconazole for example, which could imply that lower amounts of ionic liquid are needed (usually dissolved in a carrier), so the biocidal solutions effectively used could be more competitive in terms of price with already implemented wood preservatives. The use of renewable and cost-effective means of obtaining ILs could be a viable approach in lowering their price. There are reports on successful conversion (~60% yield) of D-fructose, terpenes, or alkaloids into monosubstituted imidazoles, which could further constitute building blocks for imidazolium-based ILs [128,129].

## 8. Conclusions

Ionic liquids represent a highly versatile class of organic compounds, with a broad pallet of application in the wood and paper industry. About their antifungal character, which is a relatively early domain of application, tetraalkylammonium and alkylimidazolium ionic liquids are by far the more researched ionic liquid types. Their antifungal character is inversely proportional to their surface tension, critical micellar concentration (which generally attains a minimum for a specific size of the alkyl substituents), and, most importantly, their ability to be retained on the wood’s surface. There are three commonly employed mechanisms through which ionic liquids function as biocides: through interference with the fungus’s biochemistry (cell membrane, metabolism, secreted enzymes), through chemical interaction with the secreted degrading species, and through degrading species and fungal hyphae blocking (impregnation effect). A specific ionic liquid can act through both three mechanisms, as opposed to the rest of the “classical” antifungal biocides.

The antifungal efficiency of ionic liquids depends on the type of cation, size, and position of the substituents and the choice of the anion. Due to the heterogeneity of the fungal strains tested, and the considerable variation in terms of structure, for both cations and anions, even for a particular research group, it’s hard to say which type of ionic liquid is the most efficient for a given fungal strain. On a rough analysis, ionic liquids with alkyl substituents in the range of C6–C12 seem to be more efficient as fungicides. Also, the presence of bulky substituents and heteroatoms as side-chains increase their antifungal effect. Most of the studied long alkyl chain ILs are readily retained in wood, leaching being minimal.

## Figures and Tables

**Table 1:** Basic features of wood fungal attack [21,44].

Type of Fungal Attack	Types of Wood Affected	Chemical Constituents Degraded	Degraded Wood Characteristics	Representative Fungi Examples
Brown rot	Mostly softwoods, rarely hardwoods	Cellulose, hemicellulose (live trees, timber)	Brown cubical-fracture appearance, brittle, dramatic loss in mechanical properties	*Serpula lacrymans*; *Coniophora puteana*; *Laetiporus ssp.*; *Postia placenta (Basidiomycetes)*
White rot	Mostly hardwoods, softwoods also in the case of specialized lignin attack	Lignin and hemicellulose, (specialized white rot fungi); lignin, hemicellulose and cellulose (simultaneous white rot fungi)(live trees, timber)	Bleached and fibrous-stringy appearance, loss in mechanical properties	Hemicellulose and lignin attack: *Trametes versicolor; Phanerochaete chrysosporium (Basidiomycetes); Xylaria hypoxylon (Ascomycete)* Specialized lignin attack: *Ganoderma australe; Pleurotus ssp. (Basidiomycetes)*
Soft rot	Mainly hardwoods	Cellulose, hemicellulose and to a very low amount lignin(live trees, timber, lumber)	Loss of mechanical properties, soft consistency when moist, crumbly appearance when dry	*Chaetomium ssp.; Ceratocystis ssp.; Kretzschmaria deusta. (Ascomycetes)*
Wood staining fungi	Both hardwoods and softwoods	Wood extractives and nutrients stored in wood tissue	Sapwood discoloration, blue or black/grey spots	*Ophyostoma ssp.; Ceratocystis ssp. (Ascomycetes);* *Aureobasidium pullulans; Trichoderma ssp. (Fungi Imperfecti)*

**Table 2:** Strategies employed for wood preservation.

Strategies for Wood Preservation	Principle of Action	Proposed Mechanism	Application
Biochemical	Interference with the metabolism and replication of the fungal cells [65]	The fungal cell wall sterols or polysaccharides (mannan, chitin, and α- and β-glucans) are affected	Impregnation with preservatives such as heavy metal salts, heterocyclic compounds, or long-chain (hydrophobic) ionic or non-ionic surfactants
	Interference with the secretion of fungal enzymes or their interaction with the hemicellulose and cellulose substrates [66,67]	The fungal cell wall is affected; the enzymes are inactivated or the enzymes do not recognize the lignocellulosic substrate	Impregnation with preservatives such as creosote, heavy metal salts, quaternary ammonium compounds. Wood chemical functionalization, impregnation or coating with silicates, borates, waxes or polymeric compounds (synthetic or natural) is also effective [68,69,70]. Optically-active compounds could disrupt the glucose/glucanase feedback loop, affecting the release of degrading enzymes [71].
Chemical	Blocking of the initial stage of degradation for cellulose, hemicellulose, and lignin occurring immediately after fungal colonization of wood	The highly oxidative free radicals-mediated degradation by the Fe^2+^/H_2_O_2_ system is interrupted [72]	Impregnation or coating with additives presenting antioxidant character (e.g., plant extracts) [73]
Physical	Micropores and internal voids blocking/filling	Fungal hyphae penetration is blocked; the diffusion of oxidants and enzymes into the bulk of the wood is arrested [74,75].	Impregnation or coating with hydrophobic compounds, which could impede the highly hydrophilic oxidants and enzymes spreading and coverage [76,77].
	Moisture exclusion	Hydroxyl groups in the cell wall are blocked or hindered, and free/bonded water is mobilized, impeding the enzyme penetration and degradative mechanism unfolding. Wood with a low amount of moisture is less likely to be colonized by fungi	Impregnation, thermal treatment, chemical functionalization [78,79].
